# Melanoma innervation, noradrenaline and cancer progression in zebrafish xenograft model

**DOI:** 10.1038/s41420-025-02523-8

**Published:** 2025-05-31

**Authors:** Francesca Lorenzini, Johanna Marines, Julien Le Friec, Nam Do Khoa, Maria Angela Nieto, Berta Sanchez-Laorden, Maria Caterina Mione, Laura Fontenille, Karima Kissa

**Affiliations:** 1 AZELEAD, 377 Rue du Professeur Blayac, Montpellier, France; 2 Institut de Génétique Humaine, Université de Montpellier, CNRS, Molecular Basis of Inflammation Laboratory, Montpellier, France; 3 Instituto de Neurociencias CSIC-UMH, San Juan de Alicante, Spain; 4 Department of Cellular, Computational, and Integrative Biology (CIBIO) University of Trento, Trento, Italy; 5 VBIC, INSERM U1047, University of Montpellier, Montpellier, France

**Keywords:** Cancer microenvironment, Cell biology

## Abstract

The tumor microenvironment, including the peripheral nervous system, plays a key role in regulating tumor biology, from initiation to cancer progression. Here, by modeling aggressive melanoma in larval zebrafish xenografts, we shed light on the dynamics of tumor innervation in the tumor microenvironment (TME). Axonogenesis and dendritogenesis were detected in the neurons surrounding the melanoma niche and neurogenesis was observed in the nearby population of the enteric nervous system. We also demonstrate the crucial role of noradrenaline in promoting melanoma progression with the dissemination and invasion of cancer cells to distant tissues. This zebrafish model will allow to uncover neural markers associated with melanoma progression to help in the design of innovative anti-neurogenic therapies targeting specifically the neuronal signals that regulate melanoma progression.

## Highlights

- Transplantation of human melanoma cells in 3 dpf zebrafish swim bladder allows the development of aggressive melanoma, whose cells invade the surrounding organs and migrate over distant locations.
- The presence of melanoma cells in the larval zebrafish induces morphological changes in the neurons inside the tumor niche, including increased axon length and dendritic arborization.
- Transplanted melanoma cells display direct contact with enteric neurons in the intestinal region and seem to use axons as route of cancer dissemination.
- Noradrenaline promotes melanoma cell migration and invasion in the zebrafish, resulting in melanoma progression.

## Graphical abstract

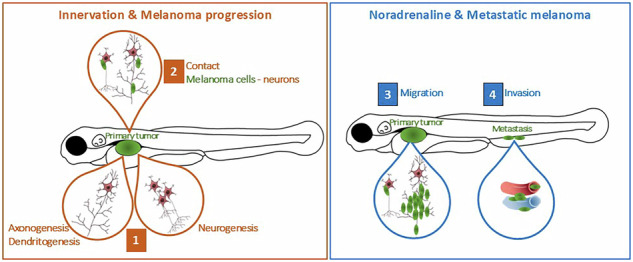

## Introduction

Cutaneous melanoma is a cancer that arises from skin melanocytes, the pigment-producing cells deriving from neural crest cells. It is the most aggressive type of skin cancer, due to its high metastatic capacity. Indeed, although it represents only 1% of all skin cancers, it causes the majority of the deaths related to skin cancers [1]. In the last 15 years, the development of targeted immunotherapies has improved the survival of metastatic melanoma patients [2, 3]. However, most patients develop resistance to therapies, because of the high plasticity of melanoma cells [4, 5] and of the presence of a very dynamic tumor microenvironment (TME).

Among the non-cancer cells that populate the melanoma TME, the functions of macrophages, vascular endothelial cells and fibroblasts have been extensively studied [6–8]. The peripheral nervous system (PNS) emerges as a novel player in cancer. It has been recently associated with carcinogenesis and cancer progression, through the release of different factors in several types of tumors. Pancreatic cancer cells can physically use the nerve fibers as a route to cancer dissemination, a mechanism that is called perineural invasion (PNI) [9, 10]. Conversely, thanks to NS plasticity, cancer cells can also impact the neural network in the TME. These changes are induced by the activation of neurogenesis and axonogenesis [11], mechanisms that are fundamental during embryonic development. Neurons can also communicate with other cells inside the tumor niche, shaping a pro-tumoral TME by inducing immunosuppression and/or angiogenesis [12, 13]. The autonomic branch of the PNS, composed of the sympathetic, parasympathetic and enteric nervous systems, is frequently associated with solid cancers [14–18]. As such, the sympathetic and parasympathetic NS have been respectively associated with prostate cancer initiation and progression [14]. Tumor innervation has also been reported for other cancer types, including gastric, skin, breast, colon and ovarian carcinomas [18]. However, little is known about the role of the PNS and innervation in the melanoma progression. It has been shown that sensory neurons counteract melanoma progression [19, 20]. However, nociceptor neurons, present in melanoma samples from patients, contribute to melanoma growth in mice by decreasing anti-tumor immune responses [21]. In addition, some preclinical studies have demonstrated the role of autonomic NS neurotransmitters like adrenaline, noradrenaline, aka catecholamines, and acetylcholine in cancer progression [14, 22]. β1, β2 and β3 catecholamine receptors, physiologically expressed in different tissues and organs, to regulate vital body functions have also been detected in the membrane of human melanoma samples and melanoma cell lines [23–25]. Activation of β-receptors seem to support cell proliferation, while inducing migration and invasion phenotypes and an EMT-like process in melanoma cells in vitro [23, 26]. Recently, a clinicopathological study demonstrated that patients developing a melanoma with high level of β2-receptor expression presented more aggressive and advanced stages of the disease. In addition, β-blockers, antagonists of β-adrenergic receptors, have positive, anticancer effects in the treatment of melanoma patients [27]. All of this supports an active role of adrenergic signaling in melanoma progression. Therefore, studying the relevance of β-adrenergic signaling and tumor innervation, both as a diagnostic and prognostic factor, as well as in the perspective of developing innovative anti-neurogenic therapies is of utmost importance. However, tumor innervation in melanoma has never been imaged in vivo in a pre-clinical model.

Xenografts in larval zebrafish have emerged as a very powerful tool to characterize the role of different components of the TME [28], thanks to the availability of many transgenic lines bearing reporters of different cells in the TME. Here, we report that a human melanoma xenograft model in the zebrafish embryo that we have recently developed [29] fully recapitulates melanoma progression to the invasive state. We show that melanoma cells were able to escape from the primary mass and disseminate to both nearby and distant locations. Using real time imaging, we further investigated the presence of tumor innervation and revealed the dynamics of tumor-induced neurogenesis and axonogenesis. The first in vivo pro-tumoral role of catecholamines was also unraveled. Altogether, our in vivo xenograft model arises as a model of choice for rapid testing of new drugs, including β-blockers, in all stages of cutaneous melanoma in a live organism.

## Results

### Melanoma xenografts in larval zebrafish recapitulates steps of an aggressive cancer

**Fig. 1:**
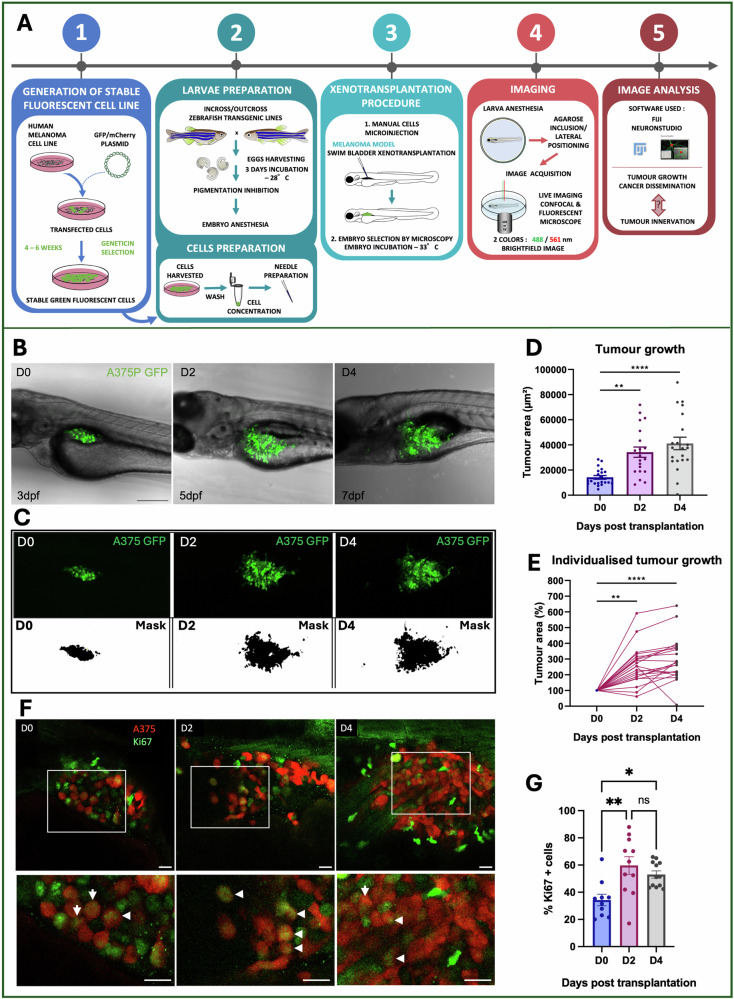
Evaluation of in vivo melanoma growth. **A** Experimental workflow of zebrafish xenograft assay. (1) Generation of melanoma fluorescent human cancer cell lines (A375); (2) Cells and larvae preparation prior to transplantation; (3) Xenotransplantation procedure; (4) Imaging and Image analysis (5). **B** Transplanted larvae with A375P GFP+ (green) are shown in the images, acquired with a confocal microscope from D0 to D4. Images derive from Z-projection (z-stack = 170-200 μm). Scale bar = 250 μm. **C** Representative xenografted melanoma tumors (green or black) are shown to explain how the analysis of tumor growth was made. Tumor growth was measured on the maximum projection of z-stack acquisitions. To determine tumor area, a threshold was applied in Fiji for each larva on D0, D2,and D4 to generate a mask. First line: maximum projection of the images on D0, D2, and D4 (z-stack = 170–00 μm). Second line: masks of the corresponding tumors, generated using Fiji software. **D**, **E** Tumor growth analysis. **D** Individualized tumor areas were normalized to D0 to monitor the percentage of the tumor growth in every single larva. **E**. The tumor area of individualized larvae was daily monitored from D0 to D4. The tumor area is expressed in μm^2^. **F** Representative xenografted melanoma tumors stained for Ki67 marker are shown to visualize proliferate A375 proliferative cells. In red A375 melanoma cells, in green Ki67+ cells. Images are single slices acquired for different xenografted larvae on D0, D2, and D4. Scale bar: 20 µm. **G** Percentage of Ki67 + A375 cells in transplanted larvae on D0, D2, and D4. Results in (**D**, **E**, **G**) are expressed as mean ± SEM, *n* = 10–21. Friedman test and ordinary one-way ANOVA were used to evaluate the significance: *P-value < 0.05, **P-value < 0.01, ***P-value < 0.001, ****P-value < 0.0001.

To evaluate the presence and role of innervation in melanoma progression, we used a novel transplantation assay, developed in zebrafish and described in Lorenzini et al. 2023 [29]. Either green or red fluorescent human malignant melanoma cell line (A375P) were transplanted in the swim bladder (SB) of 3 dpf larvae (Fig. 1A). Live-image acquisition was performed from the day of transplantation (D0) up to 4 days post transplantation (D4) (Fig. 1B, C). Melanoma cells were able to proliferate, establishing a solid tumor mass that increased its size over time (Fig. 1C–E*;* *n* = 20/21). Significant melanoma growth was observed from D0 to D2 (**P-value < 0.0061) and also from D0 to D4 with a mean tumor area of 14198.7 μm^2^ on D0 to 41159.6 μm^2^ on D4 (****P-value < 0.0001) (Fig. 1D). The tumor area was then normalized on D0 to assess the percentage of tumor growth (Fig. 1E). Melanoma xenografts were characterized by an increase of tumor area of +254% (**P-value = 0.0061) on D2 and +296% on D4 (****P-value = < 0.0001) (Fig. 1E). The Ki64 immunostaining, highlighting cells in division (Fig. 1F), demonstrated that A375 cells divide starting from D0, with the highest division rate on D2 (Fig. 1F, G*;* **P-value < 0.0021; *P-value < 0.0332). Melanoma xenografts exponentially and significantly increased their size in the first 2 dpT to then grow at a slower rate (Fig. 1D, E, G).

**Fig. 2:**
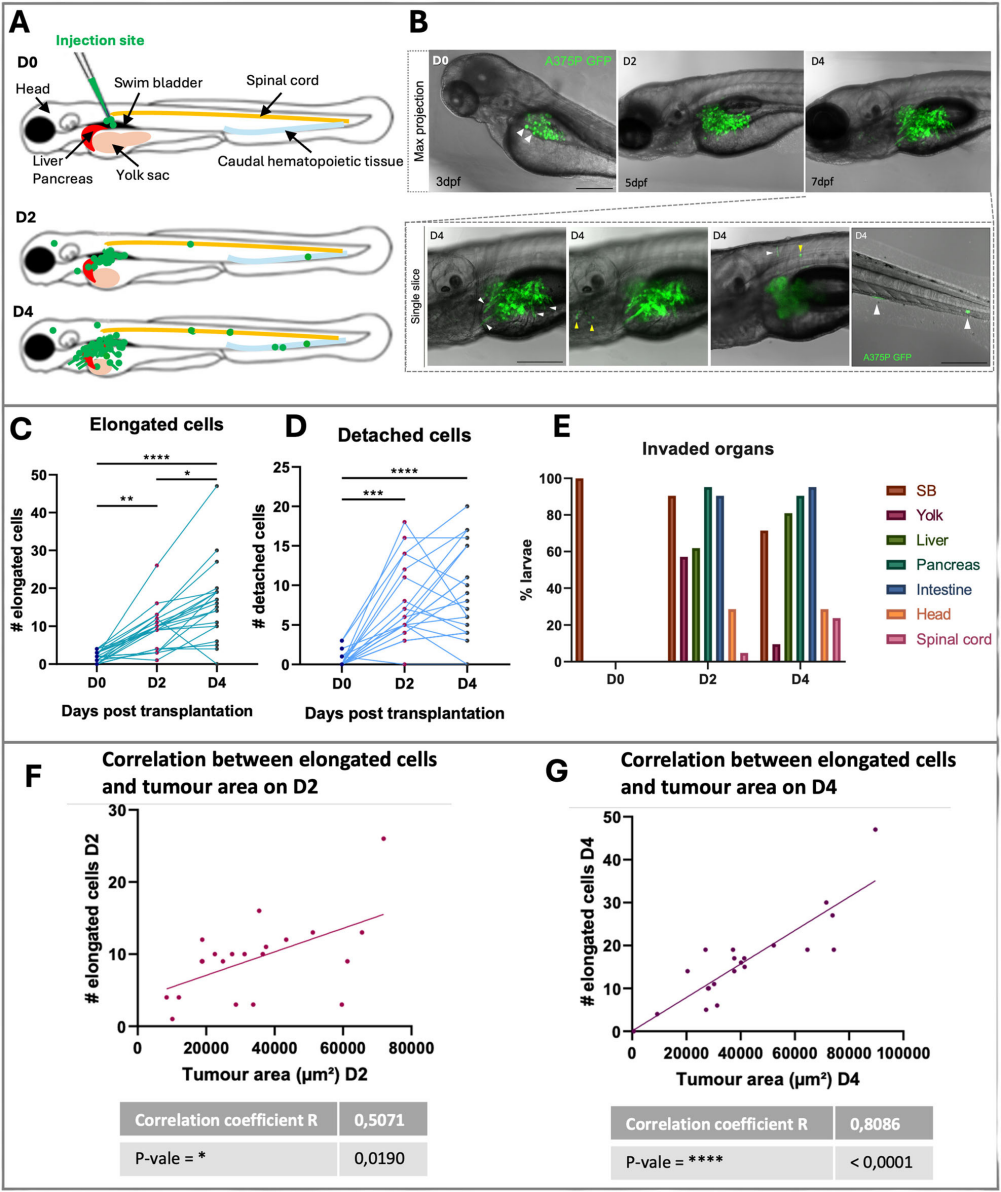
In vivo invasiveness and dissemination of A375P cells after xenografting in larval zebrafish. **A** Schematic representation of A375P cells transplanted in the swim bladder of a zebrafish larva at 3 dpf and monitoring of the tumor fate at D2 and D4. **B** Representative images of transplanted larvae with invading and migrating A375 GFP+ cells (green) on D0, D2, and D4. First line: z-projection images (z-stack = 170–200 μm). Second line: four different single slices of the same embryo acquired in the swim bladder and the caudal hematopoietic tissue (last panel) at D4. Scale bar = 250 μm. White arrowheads point elongated and disseminated cells. **C** The graph shows the quantification of elongated cells. The invasive cells were daily counted in every larva on D0, D2, and D4. **D**. The graph shows the quantification of detached cells from the primary cancer mass. The detached cells were daily counted in every larva on D0, D2, and D4. Friedman test was used to evaluate the significance in (**C**) and (**D**): *P-value < 0.05, **P-value < 0.01, ***P-value < 0.001, ****P-value < 0.0001. *n* = 21. **E** A375P cells invade the surrounding organs of the SB in larval zebrafish. The graph shows the percentage (normalized to the total number of larvae) of larvae with melanoma cells in the surrounding organs/regions near the SB on D0, D2, and D4. **F**, **G** The graphs represent the correlation between the number of elongated cells and the tumor area (μm^2^) on D2 (**E**) and on D4 (**F**). Scale bar = 250 μm. Non-parametric Spearman correlation was performed to test the possible correlation: *P-value < 0.0332, **P-value < 0.0021, ***P-value < 0.0002, ****P-value < 0.0001. *n* = 21.

We then focused on the morphology and the migratory capacity of A375P cells. The high invasive capacity of our melanoma model was assessed by counting the number of elongated and detached cancer cells from the primary tumor mass from D0 to D4 (Fig. 2A). On D0 most of the A375P cells presented a round shape and few cancer cells were characterized by ellipsoidal morphology (Fig. 2B*, white arrowheads*). On D2, but mostly on D4, several melanoma cells presented a very elongated morphology in comparison to D0 (Fig. 1B). Quantification analysis demonstrated a + 492% significant increase of the number of elongated cells on D2 (**P-value = 0.0061) and +861% significant increase on D4 (****P-value < 0.0001) in comparison to D0 (Fig. 2C). These data showed a significant increase of elongated cells also between D2 and D4 (*P-value = 0.0129), demonstrating an infiltrative phenotype.

After transplantation, there was a significant number of disseminated cells on D2 (***P-value = 0.0002) and D4 (****P-value < 0.0001) in comparison to D0 (Fig. 2A, D) in the tissues and organs surrounding the SB but also in distant locations (Fig. 2B, inset, white arrowheads, Fig. 2E).

We then studied a possible correlation between the tumor area and the number of elongated cells on D2 and D4. On D2, the correlation coefficient R was higher than 0 (*R* = 0.5071, *P-value = 0.0190), indicating a direct relation between the two parameters (Fig. 2F). This correlation was stronger on D4 (*R* = 0.8086, ****P-value < 0.0001) (Fig. 2G) indicating that the number of elongated cells presented in the larval body increases as the tumor growth.

To validate the presence of an aggressive melanoma, we performed gene expression analysis to check the immune response, hallmark of cancer [30] (Supplementary Fig. 1). The expression of the two zebrafish pro-inflammatory cytokines *il1b* [31] and *il8* [32] was tested in the larval melanoma transplants (MT), and in control larvae (CTL) injected with PBS in the SB. The analysis of the two zebrafish interleukins were significantly increased in MT in comparison to CTL, both on D0 (Supplementary Fig. 1A, C) and D2 (Supplementary Fig. 1B, D). We also compared the expression of human *IL8* in the MT on D2 and D4. Interestingly, *IL8* expression was statistically reduced on D4 in comparison to D0 (Supplementary Fig. 1E), indicating a possible reduction of the inflammatory status of the cancer niche. To compare these results with another zebrafish melanoma model, we checked the gene expression of *il1b* and *il8* in melanoma biopsies of adult *kita:ras* zebrafish [33]. *Kita:ras* biopsies had an higher *il1b* and *il8* expression in comparison to wild type skin biopsies (Supplementary Fig. 1F, G). Thus, zebrafish melanoma models seem to recapitulate the human condition of an inflammatory status. Together, our model recapitulates aspects of malignant human melanoma.

### Tumor-induced axonogenesis in melanoma xenografts

**Fig. 3:**
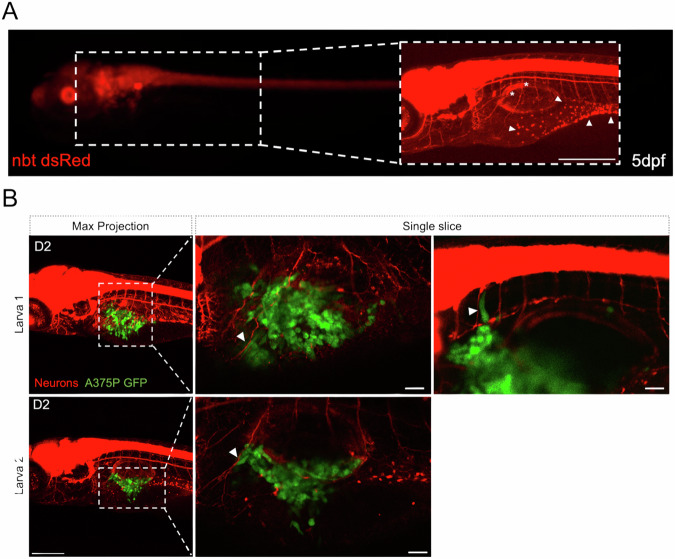
Interactions between PNS and transplanted melanoma cells in larval zebrafish. **A** Representative images of a 5 dpf *tg(nbt:dsRed)* larva, acquired with a fluorescent microscope. Zoomed area: picture of the same larvae acquired with a confocal microscope. Scale bar = 250 μm. White arrowheads = enteric neural cell bodies. White asterisks = axons. **B** Representative images of xenografted larvae with A375P cells on D2, acquired with a confocal microscope. Transplanted A375P cells are in green, neurons are in red. A375 cells seem to escape from the primary cancer mass by following the axons as a route of dissemination (white arrowheads). First column: z-projection images (z-stack = 170–200 μm) of larva 1 and larva 2. Scale bar = 250 μm. Second and third columns: zoomed images of the region around the SB of larva 1 and larva 2 corresponding to different single slices. Scale bar = 50 μm.

To visualize the presence of tumor innervation in our melanoma xenograft model, A375P cells were transplanted in Tg(*nbt:DsRed)* transgenic zebrafish larvae [34] (Fig. 3A) in which all neurons express the DsRed fluorescent protein. These neurons express high levels of AChE. Confocal microscopy revealed neurons morphological changes (Fig. 3A*, white asterisks*) compared to control embryos. Moreover, it was possible to discriminate the enteric neural cell bodies in the region of the intestine (Fig. 3A*white arrowheads*). In addition, human melanoma cells (green) started invading the surrounding tissues by interacting with neurons (red) and migrating along their axons (Fig. 3B, *white arrowheads*), in an event that resembles PNI [9, 35].

**Fig. 4:**
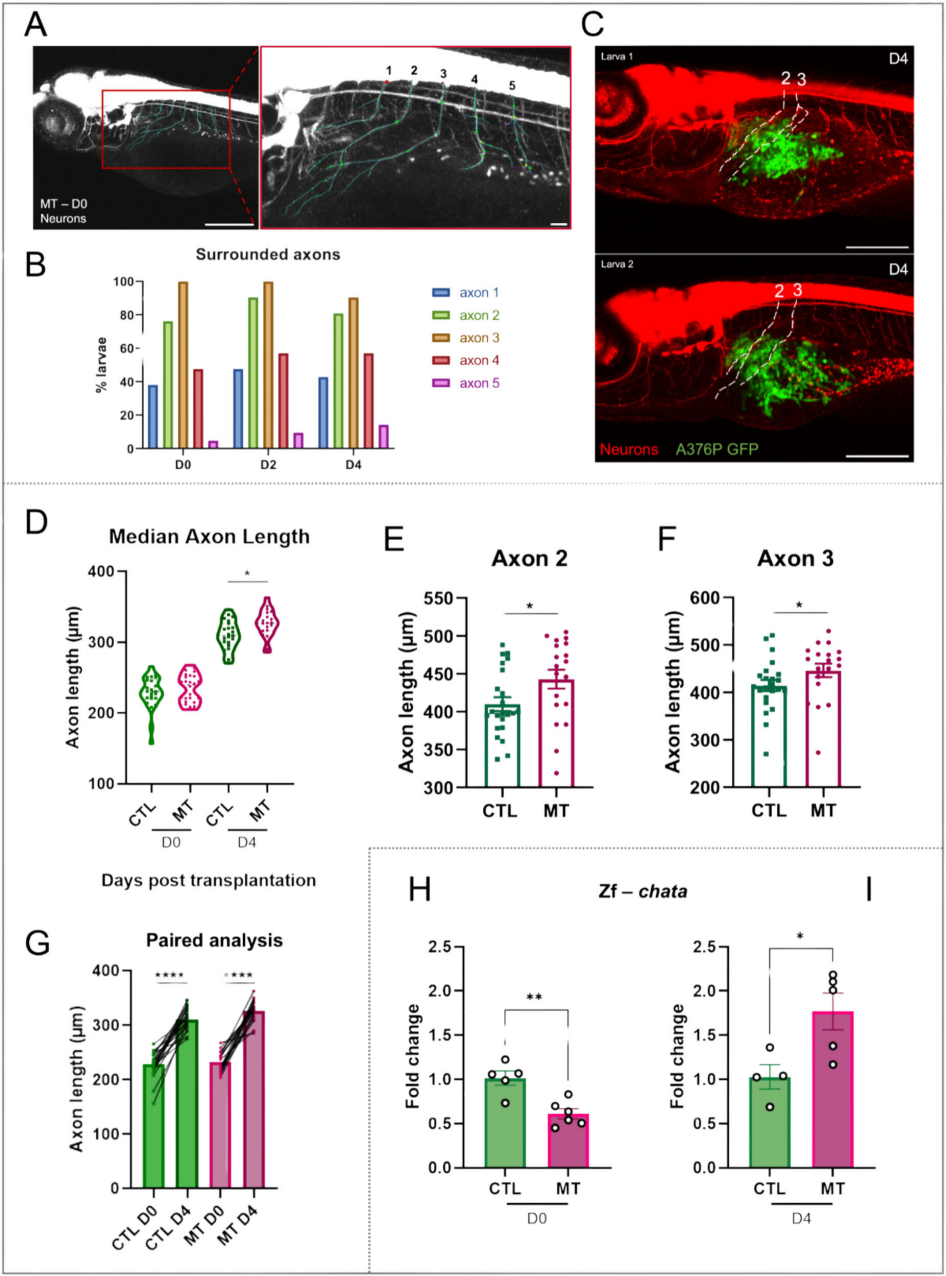
Axonogenesis in zebrafish xenograft melanoma model. **A** Representative images of a 3 dpf larva from NeuronStudio software. Five axons around the region of the SB were selected and manually traced in 3D using NeuronStudio to measure the axon length and to count the dendritic branching points. Scale bar = 250 μm/Scale bar = 50 μm. **B** The graph shows the percentage of larvae with axons 1–5 surrounding A375P cells. **C** Representative images of 7 dpf (D4) larvae with marked axon numbers 2 and 3. The images are taken using a confocal microscope. Scale bar = 250 μm. **D**–**G** Axonogenesis was observed in the xenograft melanoma model. **D** The graph shows the average length of the five axons in every single larva in every condition on D0 and D4. *n* = 19–26. The measure of the axon length was performed using the NeuronStudio software. After manually tracing the axons and the dendritic branches, the software gave a summary with the length of every axon. **E**, **F** The graphs show the axon length on D4 of the axon number 2 (**E**) and axon number 3 (**F**), the most affected axons by the presence of A375P cells. Differences among groups were analyzed by two-tailed unpaired Student’s *t* test in (**D**–**F**): *P-value < 0.05. *n* = 19–23. **G** The graph represents the paired analyses of the average of the five axons length of CTL and MT conditions on D0 and D4 as indicated. Differences among groups were analyzed by two-tailed paired Student’s *t* test in (**G**): *P-value < 0.05, **P-value < 0.01, ***P-value < 0.001, ****P-value < 0.0001. *n* = 19–22. The values in (**D**–**G**) are expressed in μm. **H**, **I** Quantitative PCR analysis of the expression of the presynaptic cholinergic gene, choline acetyltransferase (*chata*), in the CTL and MT samples on D0 and D4 as indicated. Two-tailed unpaired Student’s *t* test was performed to evaluate the significance: *P-value < 0.05, **P-value < 0.01. *n* = 4–6. Results in (**E**, **F**) and (**H**, **I**) are expressed as means ± SEM. CTL = larvae injected with PBS. MT (melanoma transplant) = larvae transplanted with A375P GFP+ cells.

To quantify potential changes of neural morphology, we focused on five axons located around the region of the nascent SB (Fig. 4A). The axons that were mostly involved in interactions with cancer cells were axons number 2 and 3 (Fig. 4). We then measured axon length and monitored the number of dendritic branches. To evaluate the axons’ growth, we measured the length of the five axons on D0 and D4 in every single larva. Then, for every larva, the average of the five axons’ lengths was calculated (Fig. 4D). On D4 we observed a significant increase in the average axons’ length in the MT larvae compared to the CTL (Fig. 4D *P-value = 0.0111) with a significant increase in length axon number 2 (Fig. 4E, *P-value = 0.0282) and 3 (Fig. 4F, *P-value = 0.0301). This is correlated with the presence of a high number of cancer cells in the environment near these axons. Measurement of other axons on D0 and D4 are reported in Supplementary Figs. 2A, 3A.

These axons belong to neurons, produce and release the neurotransmitter acetylcholine. ChAT, choline acetyltransferase, catalyzes the rate-limiting step in the acetylcholine biosynthesis. Due to zebrafish genome duplication, this enzyme is encoded by two genes, *chata* and *chatb*. However, chata is the only one expressed in early larval stage [36] as also demonstrated by our gene expression analysis.

On D0, 6hpT, there is significant decrease of *chata* expression level in MT samples in comparison to CTL (Fig. 4H, **P-value = 0.0023). This could be linked to the possible stress or neuron-injury associated with the xenotransplantation. On the contrary, on D4, we observed an increased *chata* expression in MT samples in comparison to the CTL (Fig. 4I, *P-value = 0.0265) indicating a higher activity of the neurons and other cholinergic neurons in the zebrafish larvae, such as the subpopulation of enteric neurons [37, 38].

Together, these results support the presence of axonogenesis in the TME of our larval zebrafish xenograft melanoma model.

### Tumor-induced dendritogenesis in melanoma xenografts

**Fig. 5:**
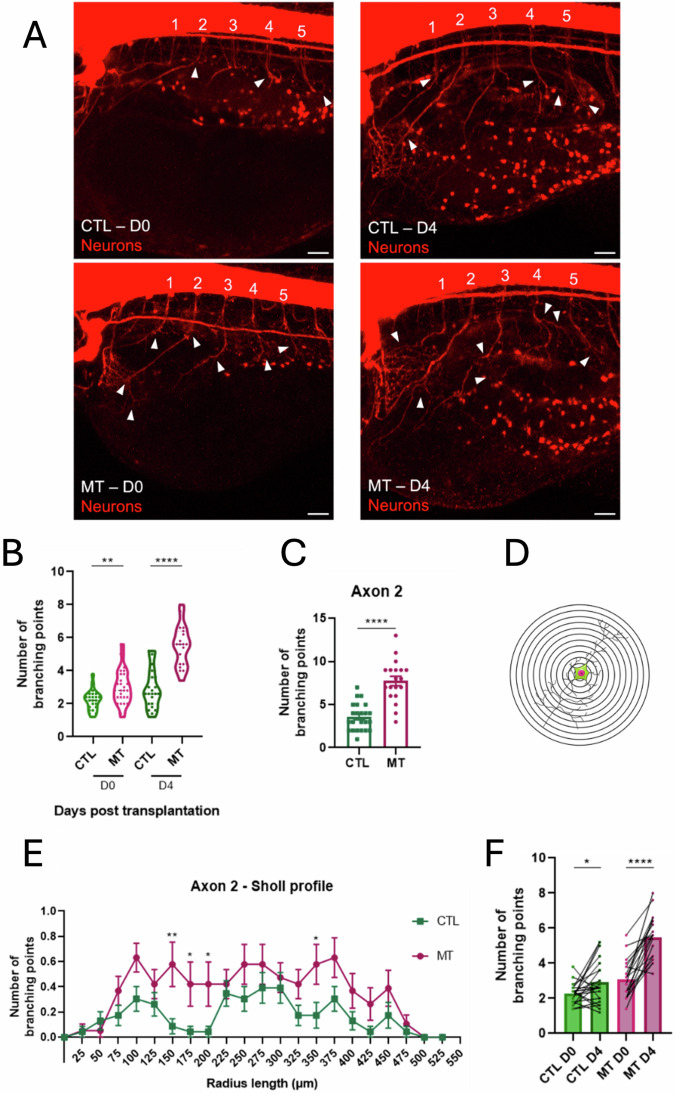
Dendritogenesis in zebrafish xenograft melanoma model. **A** Representative images of nbt CTL and MT larvae on D0 and D4. Neurons are in red. White arrowheads point the branching points. Images are taken using a confocal microscope. Scale bar = 25 μm. **B** The graph represents the average of the branching points’ number of the five axons in every larva in CTL and MT conditions on D0 and D4. *n* = 19–26. **C** The graph represents the number of branching points of the axon number 2 in every larva in CTL and MT conditions on D4. *n* = 19–23. Results are expressed as means ± SEM. Differences among groups were analyzed by two-tailed unpaired Student’s *t* test in (**A**, **B**): *P-value < 0.05, **P-value < 0.01, ***P-value < 0.001, ****P-value < 0.0001. **D** Schematic view that allows the description of the Sholl analysis, a quantitative method to study the dendritic anatomy. The picture shows concentric rings centered on the soma center of the neurons. From the center, the Sholl analysis starts counting the number of intersections of the dendrites with the different rings, reporting the distance from the soma center. The number of intersections is the number of dendritic branches. The data acquired from the analysis allow the construction of the Sholl profile. **E** The Sholl profile of axon number 2 on D4. The sholl profile is a graph that plots the number of the branching points against the radial distance from the soma center. The radial step between every ring is 25 μm. The axons analyzed in this work are from neurons, whose somas are placed in the spinal cord. Thus, the soma center was generally placed in the ventral part of the spinal cord for every neuron/axon. Differences among groups were analyzed by Mann–Whitney test: *P-value < 0.05, **P-value < 0.01. *n* = 22. Results in (**B**) and (**D**) are expressed as means ± SEM. **F** The graph represents the paired analyses of average of the five axons’ branching points of CTL and MT conditions on D0 and D4 as indicated. Differences among groups were analyzed by two-tailed paired Student’s *t* test in (**D**, **F**): *P-value < 0.05, **P-value < 0.01, ***P-value < 0.001, ****P-value < 0.0001. CTL = larvae injected with PBS. MT (melanoma transplant) = larvae transplanted with A375P GFP + cells.

We also examined the number of dendritic branching points as a readout of dendritogenesis, in the context of tumor innervation. We mapped the dendritic arborization of the five selected axons, and found that from D0, 6hpT, there was a significant increase (**P-value = 0.0033) of the average number of dendritic branching points in MT larvae in comparison to the CTL (Fig. 5A, F). The most affected axons were axons number 2, 3 and 5 (Supplementary Fig. 2B). This increase became bigger on D4 (Fig. 5A, ****P-value < 0.0001), and all 5 axons had an increased number of dendritic branching points in comparison to the CTL (Fig. 5B, Supplementary Fig. 3B). To complete the analysis of the dendritic morphology, we performed Sholl analysis (Fig. 5C) with the construction of the Sholl profile (Fig. 5D, Supplementary Figs. 2C, 3C). On D4, the MT Sholl profiles exhibited a higher increase in comparison to the CLT (Supplementary Fig. 3C) with a significant difference for axon 2 (Fig. 5D). This means that the most affected axon by the presence of human A375P cells is the number 2, both in the increase of the axon length (Fig. 4E), the increase of the number of dendritic branching points (Fig. 5B) and the Sholl profile (Fig. 5D).

As PNS developments is still ongoing in the larval stage, we performed an additional analysis from our data in which the evolution of the average of the axons’ length and dendritic branching points in every single larva on D0 and D4 was considered. Paired analysis of CTL and MT showed an increase of branching points on D4 in the transplanted embryos in comparison to the CTL (Fig. 5E; CTL: *P-value = 0.0335 VS MT: ****P-value < 0.0001), despite the presence of the developmental force. This difference is not so visible focusing on the measure of the axon length (Fig. 4G; CTL: **** VS MT: ****P-value < 0.0001). This indicates that the presence of cancer cells impacts more on the formation of new branching points, than on the promotion of axon length where the PNS developmental force is much more relevant.

### Tumor-induced neurogenesis in melanoma xenografts

**Fig. 6:**
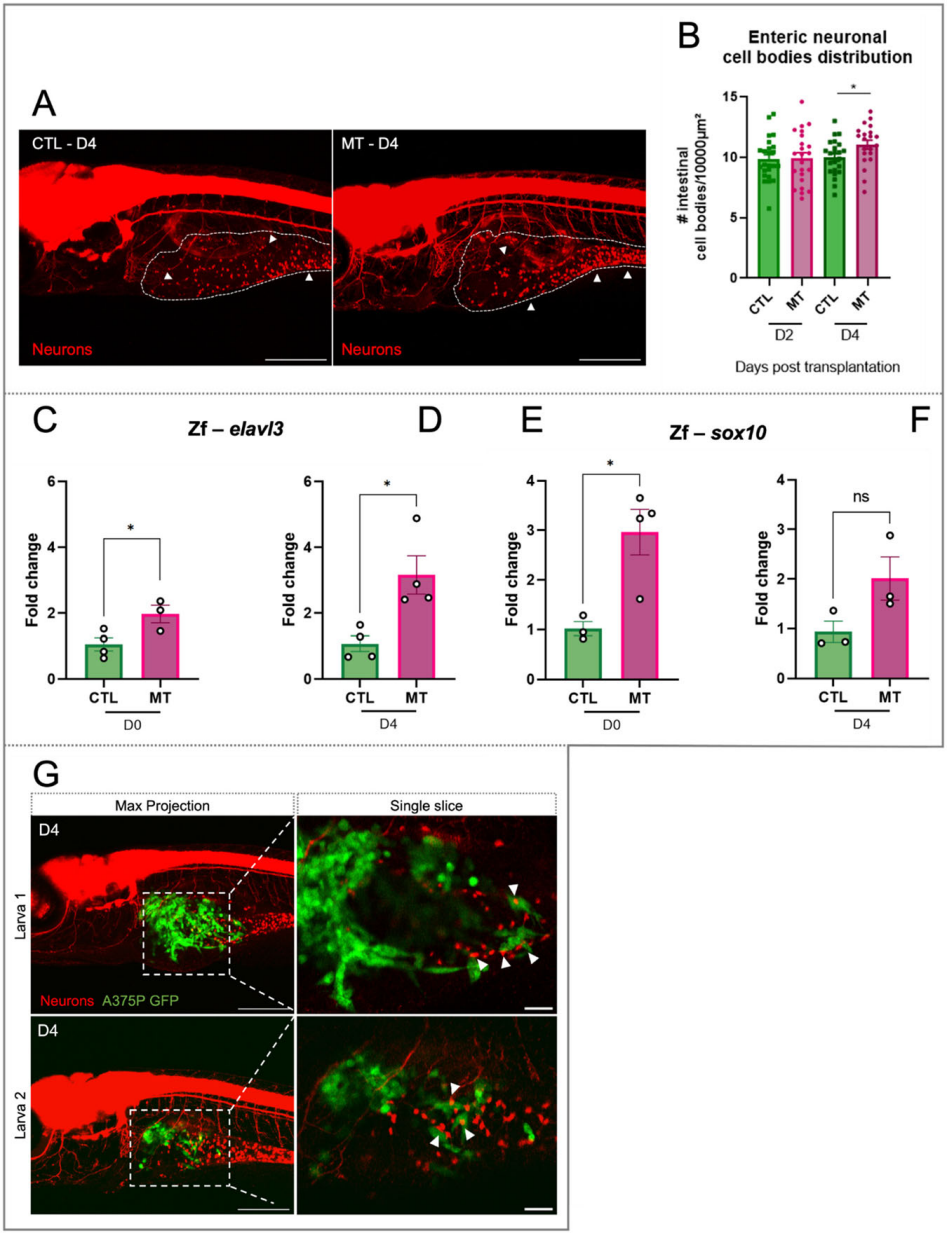
Neurogenesis in zebrafish xenograft melanoma model. **A** Representative images of *nbt* CTL and MT larvae on D4. Neurons are in red. Surrounded areas delimit the intestinal zone, white arrowheads point the enteric neural cell bodies. Images are taken using a confocal microscope. Scale bar = 250 μm. **B** The graph shows the number of enteric neural cell bodies per 10^4^ μm in CTL and MT conditions on D2 and D3. Differences among groups were analyzed by two-tailed unpaired Student’s *t* test: *P-value < 0.05. *n* = 21–24. **C**–**F** Quantitative PCR analysis of the expression of the early neurogenesis marker *elavl3* (**C**, **D**) and the NC marker *sox10* (**E**, **F**) in CTL and MT samples on D0 and D4 as indicated. Two-tailed unpaired Student’s *t* test was performed to evaluate significance: *P-value < 0.05. *n* = 3–4. Results in (**B-F**) are expressed as means ± SEM. **G** Representative images of xenografted larvae with A375P cells on D4. Images were acquired with a confocal microscope. Transplanted A375P cells are in green, neurons are in red. First column: z-projection images (z-stack = 170–200 μm) of larva 1 and larva 2. Scale bar = 250 μm. Second column: single slice of the zoomed area of the corresponding projection images. White arrowheads point the contact between enteric neural cell bodies and cancer cells. Scale bar = 50 μm. CTL = larvae injected with PBS. MT (melanoma transplant) = larvae transplanted with A375P GFP + cells.

Due to the high invasion of the intestinal tract by the A375P cells, we monitored the enteric neurons (Figs. 3A, 6A, *white arrowheads*) to visualize a possible effect of melanoma cells on this specific cell type. Quantitative in vivo image analysis demonstrated a significant increase of the number of the enteric neural cell bodies in the MT larvae in comparison to the CTL on D4 (Fig. 6A, B, *P-value = 0.0293) but not on D2. To validate this result, we quantified the expression of two genes, *elavl3* and *sox10*. *Elavl3* gene encodes for a protein that is a marker of early neurogenesis [39, 40], and consistent with an induced neurogenesis, its expression was significantly increased in xenografted larvae in comparison to the CTL (Fig. 6C, D, D0 -*P-value = 0.0378, D4 -*P-value = 0.0161). Moreover, comparing its expression from D0 and D4 in MT samples, we observed an increase of e*lavl3* expression over the time.

*Sox10* is a neural crest marker and transcription factor important for the development of enteric neural progenitors [41], Schwann cells [42] and melanocytes [43]. Its expression was higher in the xenografted larvae in comparison to the CTL, even though the result is statistically significant just on D0 (Fig. 6E, F, *P-value = 0.0170). High *sox10* expression can be associated with the enteric neurogenesis process and to an increase of the Schwann cell number in the MT larvae. The expression of *sox10* was also checked in the *kita:Ras* melanoma biopsies. In this case, the increased level of *sox10* transcript was associated with the presence of the zebrafish melanoma mass, which is absent in the wild type (CTL) biopsies (Supplementary Fig. 1H).

Interestingly, in high resolution images, we also observed contacts between A375P melanoma cells and enteric neural cell bodies (Fig. 6G*, white arrowheads*). The direct contact could mediate a reciprocal communication between the two different cell types, supporting, concomitantly, cancer invasiveness and tumor innervation through an increase of neurogenesis. Altogether these results demonstrate the engagement of the PNS in melanoma xenografts.

### Catecholamines support melanoma progression

We then characterized the role of innervation on cancer progression. Many studies have reported that the autonomous PNS is involved in cancer progression [14, 26, 44]. Focusing on malignant melanoma, in vitro and clinical data suggest that adrenergic signaling are relevant in melanoma proliferation and invasion [23–27]. To investigate this in vivo, we injected A375P cells with 1 µM noradrenaline (NA) or 0.1 µM adrenaline (AD) [45–47], mimicking the presence of catecholamines in the TME. After transplantation, the expression of the human *ADRB2* gene, that codifies for the β2-receptor was maintained from D0 to D4 at the same levels (Supplementary Fig. 4A).

**Fig. 7:**
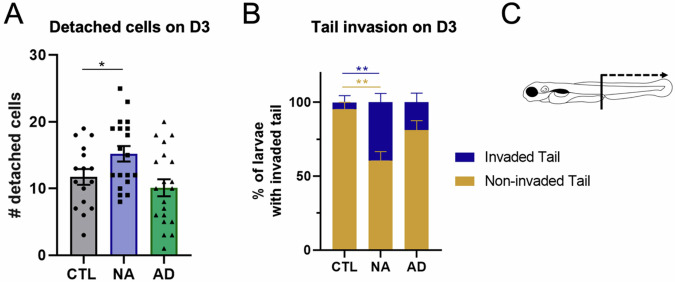
Effect of *catecholamines in xenograft melanoma model.* **A** The graph shows the quantification of detached cells from the primary cancer mass on D3. Detached cells were counted daily in larvae for every condition on D0 and D3. Larval xenografts with already disseminated cells on D0 were excluded from the analysis, to avoid considering larvae with accidentally transplanted cells in the blood circulation. Differences among treated conditions and control conditions were analyzed by ordinary one-way ANOVA: *P-value < 0.05. *n* = 16–20. **B** Graph represents the percentage of larvae with tail invaded/non-invaded by A375P cells in the three indicated conditions. Differences among treated conditions and control conditions were analyzed by two-way ANOVA: *P-value < 0.0332, **P-value < 0.0021. *n* = 19–20. **C** Schematic representation of larva where the region of the tail is indicated. Cancer cells in the tail = detached cells localized from urogenital opening until the posterior part of the CHT. NA 10 μM = larvae transplanted with A375P cells and co-injected with 10 μM of noradrenaline. AD 1 μM = larvae transplanted with A375P cells and co-injected with 1 μM of adrenaline.

Transplanted larvae were monitored using live image on D0 and D3 to assess cancer growth, cell migration and invasive capacity. Catecholamines did not increase tumor growth (Supplementary Fig. 4B) but A375P cells had a significantly higher migration capacity in the presence of NA (Fig. 7A, *P-value = 0.0370, Supplementary Fig. 4D). Moreover, catecholamines induced A375P cells delamination associated with higher invasion of the tail (Fig. 7B, C and Supplementary Fig. 4D). We observed a significantly increase (**P-value = 0.0097) of tail’s invasion by A375P cells in NA treated larvae in comparison to CTL ones (Fig. 7B) on D3. AD also increased the number of larvae with invaded tail in comparison to CTL, supporting a tendency to increased cancer aggressiveness mediated by catecholamines. Finally, by looking at the morphology of the A375P cells, we found that catecholamines’ treatments induced an increased number of elongated cells on D3; however, the results are not statistically significant (Supplementary Fig. 4C).

Together, these results demonstrate that catecholamines, especially noradrenaline, promote melanoma progression through increased cancer cell migration and invasion of distant sites.

## Discussion

In the present study, thanks to the development of an in vivo melanoma model, we characterized the impact of melanoma cells on peripheral neurons. In addition, we show the contribution of catecholamines to melanoma progression towards a more aggressive disease.

The transplantation of A375P human melanoma cells led to the development of a highly proliferative and invasive tumor, supported by an inflammatory TME. The zebrafish was shown to be an optimal niche for melanoma progression, allowing the cells to infiltrate surrounding organs and invade distant tissues. This invasion was compatible with the activation of an EMT-like process [48], also known as phenotype switching. These findings indicate that the zebrafish TME promotes aggressive melanoma behavior and needs to be further characterized.

Transplanted A375P cells in larval zebrafish migrated specifically in the CHT, a special niche in the tail, where secreted factors, such as the SDF1 [49], can attract cancer cells and sustain their survival and proliferation [50, 51]. The development of a very aggressive type of in vivo melanoma was fundamental for the following studies focused on the active function of innervation and catecholamines in melanoma progression.

Tumor innervation has become a novel hallmark of cancer initiation and progression, however only in vitro and mouse models were used to study it [9, 19–21]. Here, we demonstrated the high potential of the zebrafish model for in vivo studies of tumor innervation. Thanks to the availability of the *nbt* zebrafish transgenic line [34], we were able to follow in real time changes of the PNS in the TME of melanoma xenografts in vivo. Here, we report the response of PNS to the presence of melanoma. While in physiological conditions, neurogenesis mostly occurs during embryogenesis or in the adult enteric NS [52, 53], it is reactivated in some malignancies, including prostate cancer, gastric and colorectal carcinoma [54–57]. In our model, we confirm the presence of an active neurogenesis in the enteric NS, also supported by an increased expression of *elavl3*, an early marker of neurogenesis.

Moreover, several solid cancers are innervated by nerve fibers of the PNS, especially those arising from the autonomic NS, contributing to the formation of the TME [14, 16, 19–21]. The activation of axonogenesis and dendritogenesis processes resemble the ones happening in the peripheral nerves after injury [11]. In our model, we detected modifications of neurons morphology surrounding the melanoma mass, including increase in axonal length and in the number of dendritic branching points, generating a more complex dendritic arborization in the melanoma TME. High level of *chata* gene expression confirmed an increased cholinergic activity of the neurons but also of the cholinergic population of the enteric neurons.

In the adult PNS, changes in the neural network can be induced by Schwann cells, as they secrete growth factors that can support axonal regeneration [44]. Interestingly, cancer cells can secrete cancer-related neurotrophic factors that can similarly stimulate the growth of axons and neurites in the TME [35]. Once released, they create a molecular gradient that can promote both cancer cells proliferation and tumor innervation [35, 44]. In our model, we have shown an increase of the dendritic arborization from 6hpT, compatible with A375P melanoma cells inducing dendritogenesis. We have also observed direct interaction between A375P melanoma cells and enteric neural cell bodies, and we have observed that axonal ramifications can be used as a route of cancer dissemination (*Graphical Abstract*). Indeed, elongated A375P cells were found following axons, in a way that resembles the PNI described in innervated cancers [9, 35]. PNI is reminiscent of a well-described process, generally referred to as vessel co-option, that involves the vasculature system as a privileged route for cancer cell migration [58]. Peripheral nerve fibers and vessels grow simultaneously and following the same pathways during development [59, 60]. Thus, although cancer research has devoted many efforts to the study of vessel co-option, further experiments, including denervation strategies, will help to understand the impact of neurons on metastatic dissemination. Moreover, during PNI, in addition to neurons being attracted by cancer cells, the axons can also exert a trophic function, as nerve endings can secrete factors supporting a positive microenvironment for the survival and proliferation of cancer cells [61, 62]. In this context, our model will be useful to characterize the molecular signaling controlling melanoma innervation and can help in the design of innovative anti-neurogenic therapies.

In vitro and clinical data suggest a role of adrenaline and noradrenaline, in the regulation of melanoma progression [23, 26, 27]. In zebrafish, because the development of the sympathetic system happens in the zebrafish between 2 dpf and 8 dpf [40], we co-injected A375P melanoma cells with either adrenaline or noradrenaline, to mimic the presence of catecholamines in the human TME. Our in vivo study has demonstrated that noradrenaline promotes melanoma progression in terms of increased cancer migration and invasion.

Here, we have shown using our xenograft model that melanoma cells have an impact on the neural microenvironment. This novel zebrafish preclinical model, will allow to visualize and monitor the dynamic interactions of the tumor cells with the neural microenvironment, will help to characterize the mechanisms by which the PNS regulates melanoma biology. Finally, our model will offer the possibility to easily perform a high-content drug screening.

## Materials and methods

### Chemical compound used for in vivo experiments

Epinephrine Bitartrate (AD) and L-(−)-Norepinephrine (+)-bitartrate salt monohydrate (NA) were purchased from Merck. Before all experimental procedures fresh drug’s solutions were prepared by diluting the compounds in purified water. Zebrafish larvae were transplanted with A375P cells dissolved in PBS with either 10 uM NA or 1 uM AD. Control larvae (CTL) were transplanted with A375P in PBS.

### Animal rearing

Zebrafish (*Danio rerio*) strains were raised and maintained in the Fish Facility of AZELEAD under standard conditions [63]. Adult zebrafish were maintained on a 12/12 h light/dark cycle in a partially recirculating system and fed 3 times/day with fresh Artemia salina and dry food.

Transgenic lines (Table 1) and wildtype strains were crossed to obtain offspring for experimental procedures. Incrosses of wildtype strains and homozygous transgenic line *tg(NBT:dsRed)* were done to have offspring for cell transplantation procedure. Adult *tg(kita:GalTA4,UAS:mCherry)*^*hzm1*^; *Tg(UAS:eGFP-HRAS*^*GV12*^*)*^*io6*^ zebrafish were raised to generate adult zebrafish with melanoma tumors. All experimental procedures on zebrafish were performed in Fish Facility in accordance with the European guidelines and regulations on Animal Protection from the French Ministry of Health (F341725).

**Table 1:** List of used transgenic lines and their features.

Transgenic line	Short name	Features
*tg(nbt:dsRed)*	Nbt	Nbt drives the expression of dsRed in all the neurons.
Et(*kita:GalTA4,UAS:mCherry)*^*hzm*1^ ZDB-ALT-090702-3	Kita:Gal4	Driver line expressing the trans-activator GAL4 under the kita promoter. GAL4 activates UAS promoter that drives the expression of mCherry in the notochord. Thus Kita drives mCherry expression in the notochord [33].
*Tg(UAS:eGFP-HRAS*^*GV12*^*)*^*io006*^ ZDB-ALT-090702-2	UAS:Ras	Responder line expressing the human mutated form of HRAS oncogene. This gene hosts the GV12 point mutation and it is fused with eGFP. Its expression is mediated by the binary GAL4/UAS system. Only in presence of GAL4, that activates UAS, the oncogene is expressed [33].
Et(*kita:GalTA4,UAS:mCherry*)^hzm1^; *Tg(UAS:eGFP-HRAS*^*GV12*^*)*^*io6*^	kita:ras	Double transgenic lines obtained by crossing of Kita:Gal4 and UAS:Ras lines. Kita drives the expression of mCherry and HRAS tagged with eGFP, inducing hyperpigmentation in zebrafish larvae and melanoma formation in adult zebrafish.

### Cell culture maintenance

Human primary melanoma cell line, A375P, was cultured in Dulbecco’s modified Eagle’s medium (DMEM) (Eurobio scientific) supplemented with 1% l-glutamine 200 mM (Eurobio scientific), 1% Penicillin/Streptomycin (Eurobioscientific #CABPES01-0U) and 10% fetal bovine serum (FBS) (Eurobio scientific) at standard conditions of 5% CO2, at 37 °C. Cells were cultured in 100 mm Petri dish (Corning) and split twice a week when they reached 80–90% confluency.

### Generation of stable fluorescent human cancer cell lines

To obtain A375P-eGFP+ cells and A375P-mCherry +, A375P cells were stably transfected with either sfGFP-N1 or mCherry2-N1 plasmids as described below. sfGFP-N1 (Addgene plasmid #54737) and mCherry2-N1 (Addgene plasmid # 54517) plasmids were gift from Geoffrey Waldo and Michael Davidson. One day before transfection, cells were plated in 6 well plates. sfGFP-N1/mCherry2-N1 purified plasmid (4 μg) was transfected using 2.5 M CaCl2 reagent. 24 h after transfection, complete DMEM medium was replaced. eGFP+ cells were selected using Geneticin (800- 1000 μg/mL) diluted in DMEM medium for 4-6 weeks before starting in vivo experiments.

### Embryo and cell preparation prior to xenotransplantation

Embryos were initially maintained at 28 °C at maximum density of 50 embryos per Petri dish in fish water supplemented with 0.0002% methylene blue (Sigma) as an antifungal agent. After 24 h, embryos were placed in fish water containing 200 μM Phenylthiourea (PTU) to prevent embryo pigmentation and allow fluorescent imaging acquisition.

Fluorescent human melanoma A375P-eGFP + /mCherry+ cells were harvested from a 100 mm Petri dish the day of xenograft transplantation. Cells were washed with phosphate-buffered saline (PBS) and detached using Trypsine-Versene EDTA (Eurobio scientific) at 37 °C for 5 min. Trypsine-Versene was inactivated by complete DMEM medium, and cells were centrifuged at 1000 rpm for 5 min and then washed with PBS. In some experiments, to study the in vivo role of catecholamines on melanoma progression, catecholamines (either 10 µM NA or 1 µM AD) were added to the PBS. Finally, cells were resuspended in 50–100 μL PBS to ensure highly concentrated cell preparation.

### Xenotransplantation of human cancer cell lines

Borosilicate glass capillaries (O.D.: 1 mm, I.D.: 0.75 mm, Sutter Instrument) were pulled using glass micropipette puller (Sutter Instrument). Capillaries were filled with 10 μL of cells suspension. Injection was performed under a stereomicroscope (Leica M80 Stereo zoom microscope) using a micromanipulator (Narishige) and the oil manual microinjector (Cell Tram Vario Eppendorf). Before xenografting, 3 dpf larvae were anesthetized in 0.16 mg/ml PBS/Tricaine (MS-222). 200 to 400 melanoma cells were transplanted into the nascent swim bladder. Control larvae were injected with PBS in the same region. Larvae with fluorescent tumor mass in the swim bladder were selected and isolated in a single well of 24-well plates. Transplanted embryos were maintained at 33 °C in fish water containing PTU.

### Whole-Mount Immunofluorescence

The day of transplantation (D0), two days after transplantation (D2) and 4 days post transplantation (D4) zebrafish larvae were euthanized with tricaine (0.32 mg/ml MS-222) and fixed in 4% (v/v) formaldehyde (Sigma-Aldrich) in PBS for 2 h at room temperature. Fixed transplanted larvae were either immediately processed or transferred to 100% methanol for long-term storage. Zebrafish xenografts were rehydrated by immersion in solutions with a decreasing concentration of methanol (75%, 50%, 25%) and washed with PBS–Triton 0.1% (w/v). They were then incubated for 5 min in distilled water and in ice-cold acetone at −20 °C for 15 min. After washing with PBS–Triton 0.1%, zebrafish xenografts were blocked for 1 h at room temperature to prevent unspecific binding of the antibodies with the buffer prepared in PBS (5% NGS, 0.1% Triton, 0.001% Sodium Azide). Ki67 primary antibody (rabbit, Abcam, ab15580, 1:100) was incubated for 1 h at room temperature followed by overnight incubation at 4 °C. Zebrafish xenografts were washed with PBS-Triton 0.1% and then incubated with the secondary antibody (Alexa goat anti-rabbit 488, 1:250) for 1 h at room temperature followed by overnight incubation at 4 °C. After staining, zebrafish xenografts were mounted in low melting point agarose for confocal imaging.

### Live-image acquisition

Transplanted larvae were individualized and imaged the day of transplantation (D0), two days after transplantation (D2), and either 3 (D3) or 4 days post transplantation (D4), according to the biological question of the experimental procedure. D0 corresponds to the day of transplantation with a technical delay of about 6 h between the transplantation procedures and the image acquisition. For image acquisition, every larva was anaesthetized and positioned laterally inside a single well of a 7-position mold (designed by AZELEAD) covered with fish water containing Tricaine.

To study tumor progression and tumor innervation, live images were acquired with LSM 510 Meta Zeiss confocal microscope (Objective: 10X/1). 488 and 543 nm lasers were used with appropriate filters. Images were taken with 5.7 μm z-step interval to a total Z-stack of 170-200 μm depending on tumor size.

To study the effect of catecholamines on tumor growth and tumor invasiveness, images were acquired using the Zeiss automated microscope system Celldiscoverer 7 (CD7) (Objetives: ×5/1 and ×20/0.5). Images were acquired in z-stack mode inside a total range of 250 μm with a z-step interval of 5 μm. 470 nm LED was used to acquire green fluorescence.

Stained zebrafish xenografts were acquired with Leica TCS SP8 confocal microscope (Objective: 20X/1). 405, 488, and 561 nm lasers were used with appropriate filters. Images were taken with 5 μm z-step interval to a total Z-stack of 100–150 μm depending on tumor size.

### Image analysis

Images were processed using Fiji and NeuronStudio software. Maximum projections were generated using either Fiji software or ZEN software for the images acquired using the CD7 microscope. Melanoma growth and evolution were followed thanks to green fluorescence of A375P melanoma cancer cells. To analyze tumor growth, the maximum projection of every image was used. To determine tumor area, a threshold was applied in Fiji for each larva on D0, D2 and D3/D4 to generate a mask. The tumor area was then normalized on the day of transplantation (D0) to assess the percentage of tumor growth.

To characterize the migratory and invasive potential of melanoma cancer cells in vivo, disseminated cancer cells from the primary mass and elongated cells were manually counted on Fiji moving through the entire Z-stack. To check the presence of cancer cells in the caudal hematopoietic tissue (CHT), A375P cells were manually counted from the urogenital opening till the posterior part of the CHT.

To study tumor innervation, NeuronStudio software was used to manually trace in 3D five selected axons, named from 1 to 5, that located all around the region of swim bladder.

The length of major axons and the number of branching points were measured and counted. To describe the dendritic anatomy, Sholl analysis was performed [64]. Sholl analysis counts the number of the intersections of the dendrites with different imaginary rings, reporting the distance from the soma of the neurons, considered as the center. The number of intersections is the number of the dendritic branches. The data acquired from the analysis allow the construction of a graph called Sholl profile.

To quantify Ki67 marker, all cancer cells and the Ki67 + cells were manually counted using Fiji software to get the percentage of cells positive to the proliferative marker. The images were prepared using the Fiji software by adjusting brightness and contrast.

### Zebrafish biopsies

Adult wild type and *kita:ras* fish were sacrificed by anesthetic overdose (0.32 mg/ml MS-222) and immediately put on a homemade sectioning stage containing dry ice under a stereomicroscope (Leica M80 Stereo zoom microscope). Using scalpel and forceps, dark melanoma biopsies from *kita:ras* fish were dissected. Melanoma came from either the central body or the tail of adult fish. As control, biopsies from wild type fish were obtained in the same way. Melanoma and wild-type biopsies were immediately snap frozen using dry ice after dissection. Samples were stored in 2 ml Eppendorf in liquid nitrogen.

### RNA extraction, cDNA synthesis and qPCR—zebrafish samples

Transplanted larvae (MT) with A375P cells and control larvae (CTL) injected with PBS on D0 and D4 were anesthetized (1x MS-222). Then larvae were beheaded under the stereomicroscope with a sterile scalpel. 10 beheaded larvae were grouped for every condition (CTL D0, MT D0, CTL D4 and MT D4) in 2 ml Eppendorf and snap frozen in dry ice. The samples were then stored at −80 °C until RNA extraction procedure.

Same RNA extraction was done on larvae samples and on adult zebrafish biopsies. RNA extraction was performed using NucleoSpin RNA purification kit (MACHEREI-NAGEL), according to the manufacturer’s protocol. At the final step, RNA was eluted in 30 μl RNase-free water to increase its concentration. The quantity and quality of RNA were assessed through NanoDrop 2000c (Thermo Scientific). Each sample was stored at −80 °C. 400 ng of total RNA was retrotranscribed to cDNA using SensiFAST^TM^ cDNA Synthesis Kit (Bioline), according to the manufacturer’s protocol. Quantitative PCR reaction mix was prepared using 2x Fast Q-PCR Master Mix (SYBR, no ROX, SMOBIO), according to the manufacturer’s protocol. cDNA was diluted 1:4 with nuclease-free water. qPCR reactions were performed on a CFX96 Real-Time PCR Detection System (Bio-Rad) machine to amplify the cDNA of human and zebrafish genes of interest. Primers were designed with Primer 3 program and were purchased from Eurofins Genomics; the sequences are reported in Table 2. The primers’ specificity was tested checking the melting curves. The analysis of gene expression is conducted using the ΔΔCt mathematical methodology on Microsoft Excel program.

**Table 2:** List of the qPCR primer sequences.

Gene name	NCBI Gene-ID	Primer sequence (5’-3’) Forward	Primer sequence (5’-3’) Reverse
Zf - elavl3	30732	GGGGAAATCGAGTCCTGCAA	GAATCTGAAGCGCTGGGTCT
Zf - chata	100170938	CGCTGTTTGTGTCTGGTGTG	ACTCGTCCCTCTTGAAAGCG
Zf - chatb	103171573	GGACCAGACAGAAACGGAGG	GCCGAGCGGATGATTTCAAC
Zf - sox10	140616	TGAACGAGACGGATAAGCGG	ATGTATACGATGGCGCTGCA
Zf - il1b	405770	TCATCATCGCCCTGAACAGA	CATGTCCAGCACCTCTTTTTCTC
Zf - il8	100002946	GACAGGTCTTTTGGCAGAGG	CTGGAGTCTCGGGTCATCC
Zf - rps11	406686	ACAGAAATGCCCCTTCACTG	GCCTCTTCTCAAAACGGTTG
Hs - ADRB2	154	CCACCAGGAAGCCATCAACT	GGACACGATGGAAGAGGCAA
Hs - IL1B	3553	CCAGCTACGAATCTCCGACC	GGGAACTGGGCAGACTCAAA
Hs - IL8	3576	GTGCAGTTTTGCCAAGGAGT	TTATGAATTCTCAGCCCTCTTC
Hs - GAPDH	2597	GTGAAGGTCGGAGTCAACG	GGTGAAGACGGCCAGTGGACTC

### Statistical analysis

Statistical analyses were conducted using GraphPad Prism 8 software. Results were shown as mean ± SEM (Standard Error of the Mean), percentage mean ± SEM and in violin plots (center lines, medians; box limits, second and third quartiles). Differences among groups were analyzed by two-tailed unpaired/paired Student’s *t* test, non-parametric test, One-way/Two-way ANOVA and Friedman test, as indicated in the figure legends. According to the statistical test, significance was set at either *p*-value < 0.05 or *p*-value < 0.0332, as indicated in the figure legends.

## Supplementary information

Supp Figures
