# Supplementary material for: Melanoma innervation, noradrenaline and cancer progression in zebrafish xenograft model
Source: Cell Death Discov. 2025 May 31;11:260. doi: 10.1038/s41420-025-02523-8 (PMC12126511; doi:10.1038/s41420-025-02523-8)
Supplement: Supplementary file 1 — Supp Figures [file 41420_2025_2523_MOESM1_ESM.docx]

**Supplementary Figures**

**Sup. Fig. 1: *Gene expression analysis of pro-inflammatory markers and the NC marker sox10.***

**
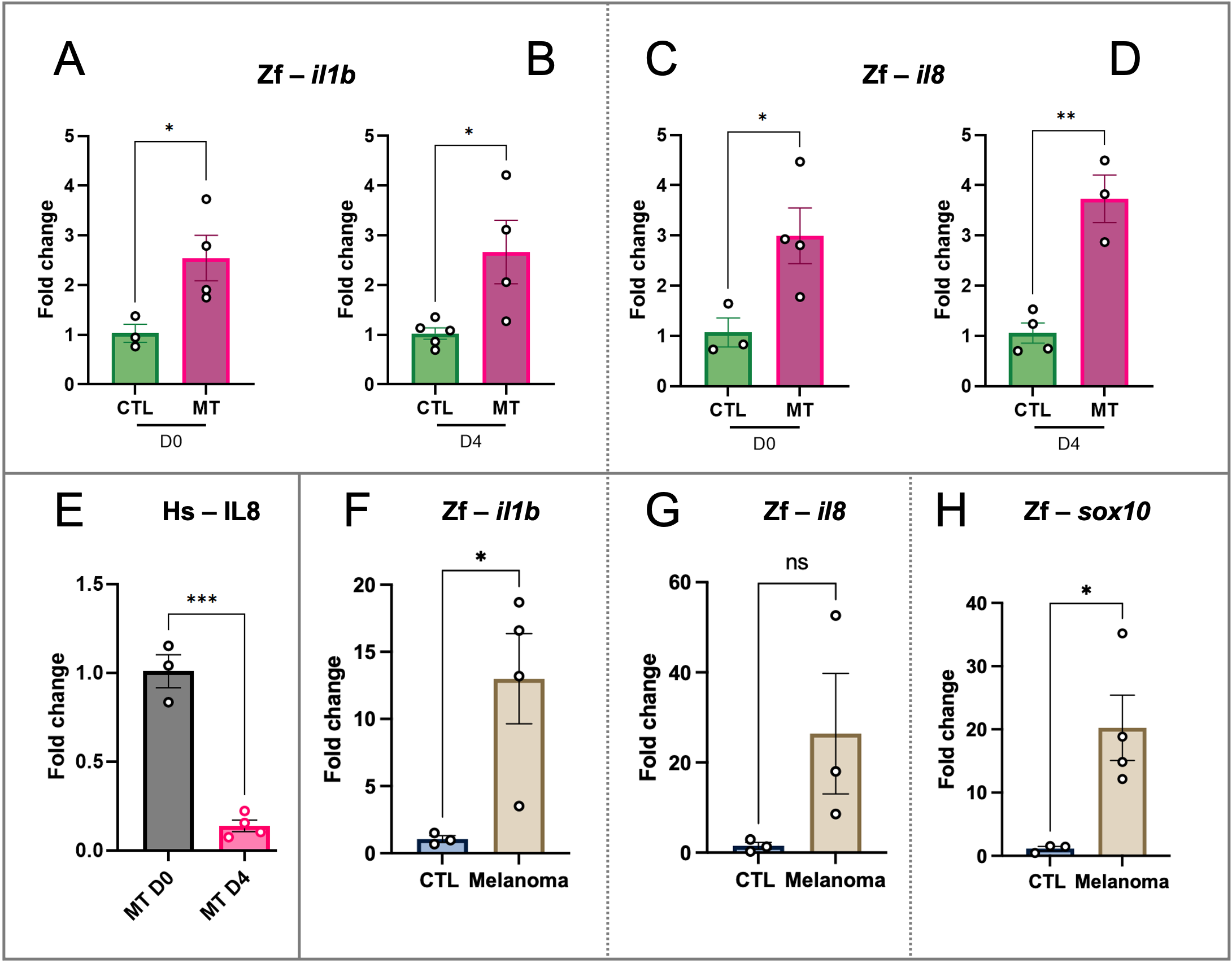
**

**A-E.** Quantitative PCR analysis of the expression of zebrafish and human pro-inflammatory markers *il1b* (**A-B**) and *il8* (**C-D, E**) in CTL and MT samples on D0 and D4 as indicated.

CTL = larvae injected with PBS

MT (melanoma transplant) = larvae transplanted with A375P GFP+ cells.

**F-H**. Quantitative PCR analysis of the expression of zebrafish pro-inflammatory markers *il1b* (**F**) and *il8* (**G**) and the NC marker *sox10* (**H**) in CTL and melanoma biopsies from respectively adult wild type and *kita:Ras* zebrafish.

CTL = body biopsies of wild type adult zebrafish

Melanoma = melanoma biopsies from adult *kita:ras* adult zebrafish

Results (**A-H**) are expressed as means ± SEM. Two-tailed unpaired Student’s t test was performed to evaluate the significance: * P-value <0,05, ** P-value <0,01, *** P-value <0,001. n=3-4.

**Sup. Fig. 2: *Complete tumour innervation analysis on D0.***


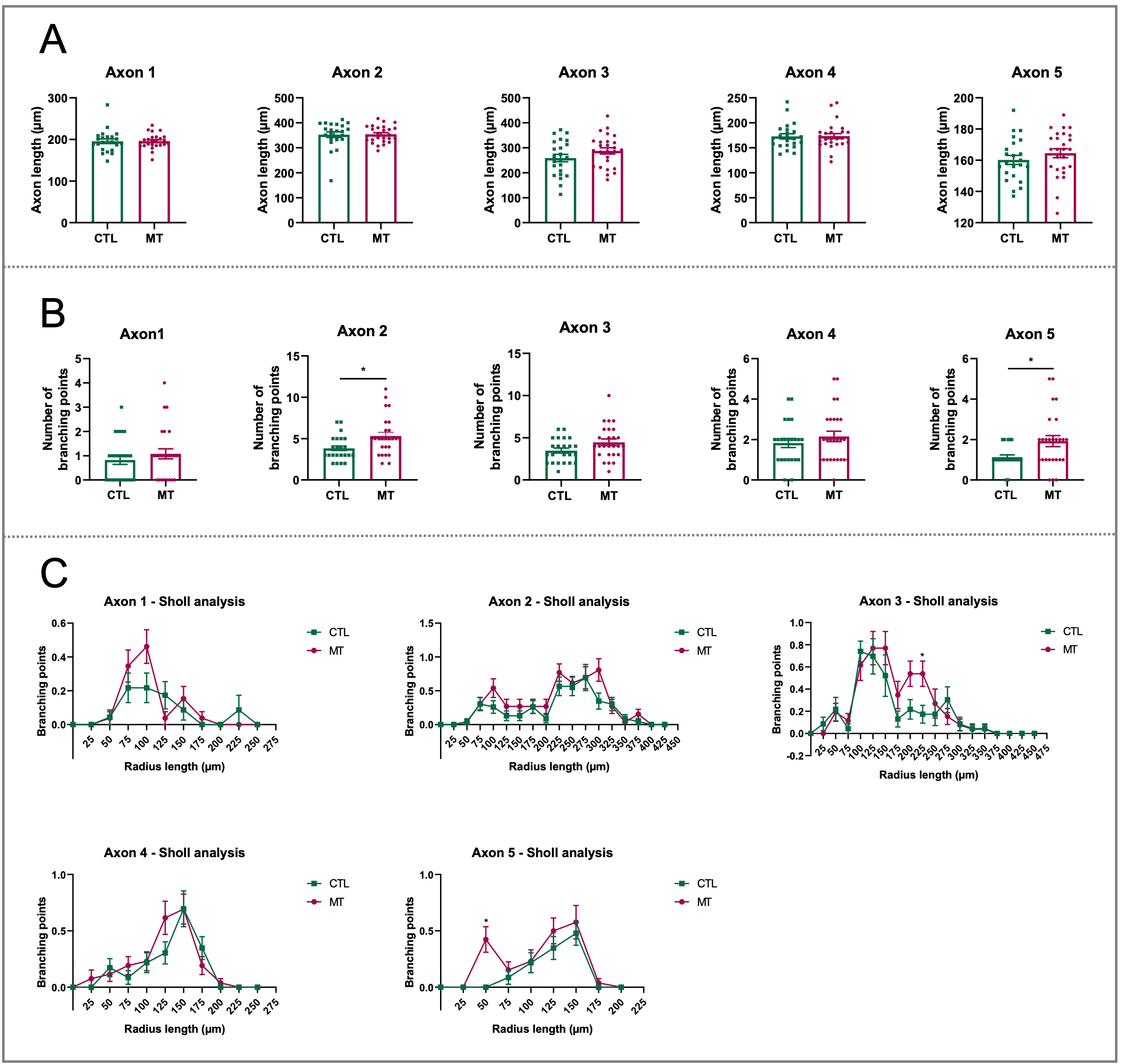


**A.** The graphs show the axon length on D0 of the axon number 1, 2, 3, 4 and 5 of CTL and MT larvae as indicated. The axon length is expressed in μm.

**B.** The graphs represent the number of branching points on D0 of the axon number 1, 2, 3, 4, and 5 of CTL and MT larvae as indicated.

Differences among groups (**A-B**) were analysed by two-tailed unpaired Student’s t test: * P-value<0,05, ** P-value <0,01.

**C**: Sholl profiles of axon number 1, 2, 3, 4, and 5 on D0 of CTL and MT larvae. Differences among groups were analysed by Mann-Whitney test.

Results are expressed as means ± SEM. n=23-26.

CTL = larvae injected with PBS

MT (melanoma transplant) = larvae transplanted with A375P GFP + cells.


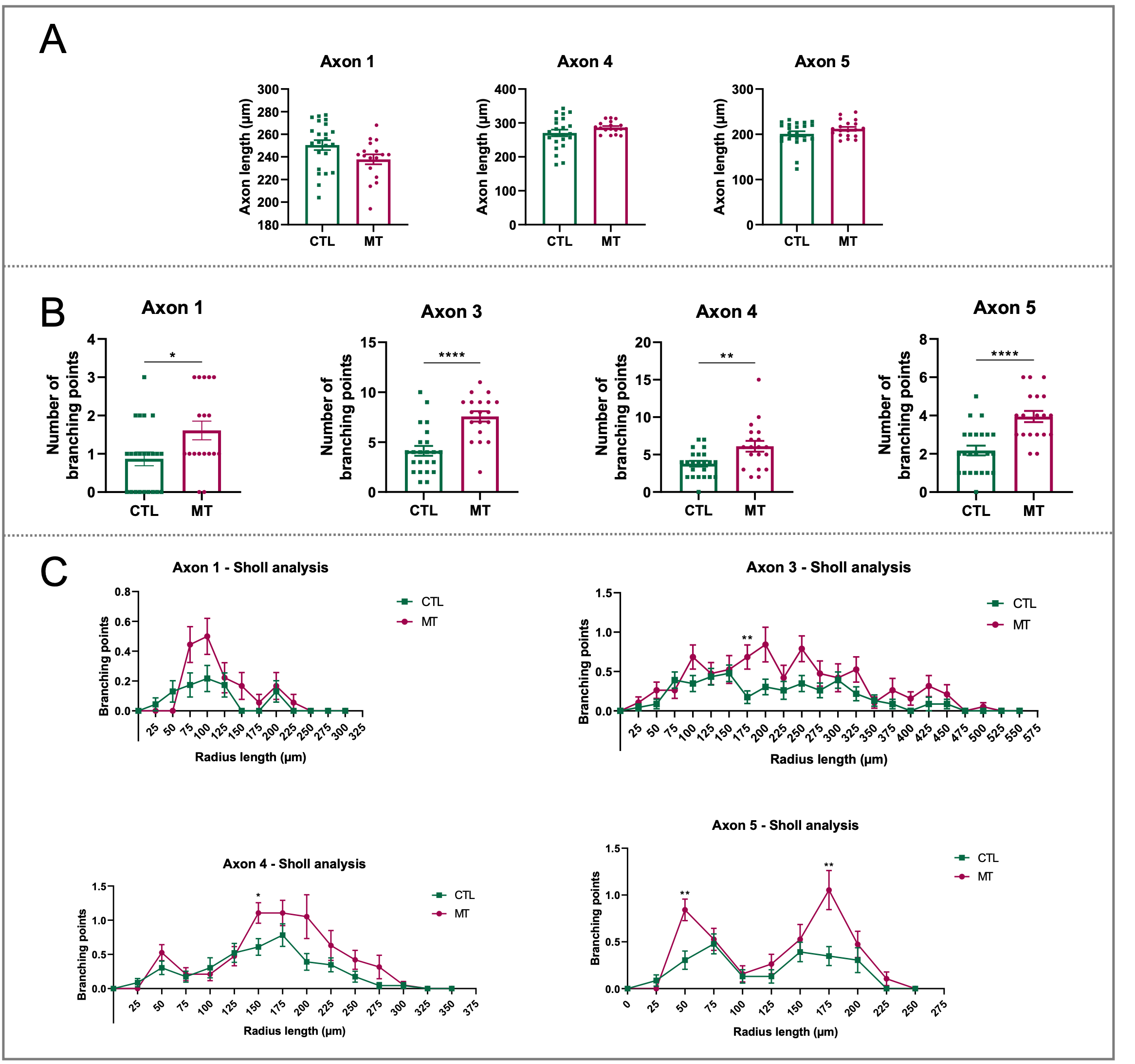


**Sup. Fig. 3:** ***Complete tumour innervation analysis on D4.***

**A.** The graphs show the axon length on D4 of the axon number 1, 4 and 5 of CTL and MT larvae as indicated. The axon length is expressed in μm.

**B.** The graphs represent the number of branching points on D4 of the axon number 1, 3, 4, and 5 of CTL and MT larvae as indicated.

Differences among groups (**A-B**) were analysed by two-tailed unpaired Student’s t test: * P-value<0,05, ** P-value <0,01.

**C**: Sholl profiles of axon number 1, 3, 4, and 5 on D4 of CTL and MT larvae. Differences among groups were analysed by Mann-Whitney test.

Results are expressed as means ± SEM. n=18-23.

CTL = larvae injected with PBS

MT (melanoma transplant) = larvae transplanted with A375P GFP + cells.

**
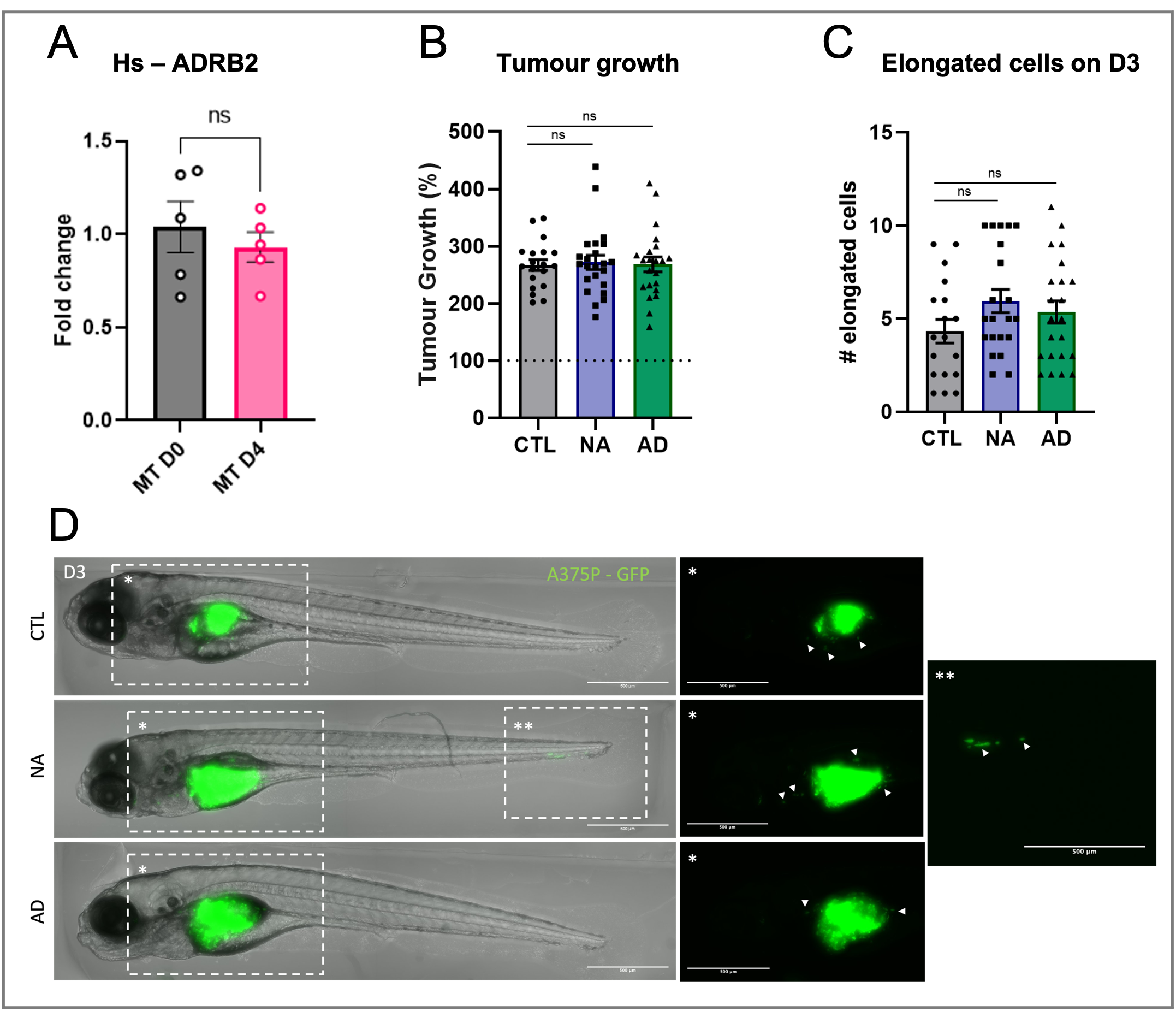
**

**Sup. Fig. 4: *Role of catecholamines in xenograft melanoma model.***

**A**. Quantitative PCR analysis of the expression of human beta-adrenergic receptor 2 (*ADRB2*) in larvae transplanted with A375P cells on D0 and D4. Two-tailed unpaired Student’s t test was performed to evaluate the significance. n=5.

**B**. Tumour growth analysis upon co-injection with catecholamines. The tumour area of individualized larvae was monitored from D0 to D3. The values are expressed as percentage of tumour growth, they are normalized on the tumour area of D0 for every separated condition. The tumour area is expressed in μm^2^. n= 19-23.

**C**. The graph shows the quantification of elongated cells on D3. Elongated cells were manually counted in larvae for every condition on D3. n=18-24.

Differences among treated conditions and control condition (**B-C**) were analysed by ordinary one-way ANOVA. Results are expressed as means ± SEM.

**D**. Representative images of transplanted larvae with A375P cells (green) co-injected with PBS (CTL), 10 μM noradrenaline (NA) and 1 μM adrenaline (AD). First column: z-projection of full larvae. Dashed rectangles point area with detached cells that are zoomed on the following images. Second/third columns: zoomed z-projection of the selected areas where detached cells are present. Scale bar = 500 μm.

CTL = larvae transplanted with A375P cells and co-injected with PBS.

NA 10 μM = larvae transplanted with A375P cells and co-injected with 10 μM of noradrenaline.

AD 1 μM = larvae transplanted with A375P cells and co-injected with 1 μM of adrenaline.
